# Mining cholesterol genes from thousands of mouse livers identifies aldolase C as a regulator of cholesterol biosynthesis

**DOI:** 10.1016/j.jlr.2024.100525

**Published:** 2024-02-28

**Authors:** James A. Votava, Steven V. John, Zhonggang Li, Shuyang Chen, Jing Fan, Brian W. Parks

**Affiliations:** 1 Department of Nutritional Sciences, University of Wisconsin-Madison, Madison, WI, USA; 2 Morgridge Institute for Research, Madison, WI, USA

**Keywords:** WGCNA, cholesterol, triglycerides, lipid metabolism, ALDOC, RDH11, ECHDC1

## Abstract

The availability of genome-wide transcriptomic and proteomic datasets is ever-increasing and often not used beyond initial publication. Here, we applied module-based coexpression network analysis to a comprehensive catalog of 35 mouse genome-wide liver expression datasets (encompassing more than 3800 mice) with the goal of identifying and validating unknown genes involved in cholesterol metabolism. From these 35 datasets, we identified a conserved module of genes enriched with cholesterol biosynthetic genes. Using a systematic approach across the 35 datasets, we identified three genes (*Rdh11*, *Echdc1*, and *Aldoc*) with no known role in cholesterol metabolism. We then performed functional validation studies and show that each gene is capable of regulating cholesterol metabolism. For the glycolytic gene, *Aldoc*, we demonstrate that it contributes to de novo cholesterol biosynthesis and regulates cholesterol and triglyceride levels in mice. As *Aldoc* is located within a genome-wide significant genome-wide association studies locus for human plasma cholesterol levels, our studies establish *Aldoc* as a causal gene within this locus. Through our work, we develop a framework for leveraging mouse genome-wide liver datasets for identifying and validating genes involved in cholesterol metabolism.

Blood and cellular cholesterol levels are tightly regulated through complex machinery that regulates biosynthesis, esterification, uptake, and export (1). While essential for normal physiology, dysregulation of cholesterol metabolism contributes to common metabolic diseases, such as nonalcoholic fatty liver disease and cardiovascular disease (2). In the case of cardiovascular disease, elevated plasma lipid levels, including low density lipoprotein cholesterol (LDL-C) are a key risk factor (3). Given the strong heritability of plasma lipids in humans, there has been significant research dedicated to identifying the underlying genetic factors that contribute through the use of genome-wide association studies (GWAS) (4). Through these studies, geneticists have identified more than a hundred loci associated with plasma levels of total cholesterol (TC), LDL-C, high density lipoprotein cholesterol (HDL-C), and triglycerides (5, 6, 7). Despite the tremendous success of GWAS in identifying loci, a major goal in the post-GWAS era is to translate this genetic data and understand the biological pathways through which the underlying causal genes operate (8, 9, 10).

In humans and mice, the liver is a major regulator of systemic cholesterol and triglyceride metabolism through sensing, uptake, de novo biosynthesis, and extrahepatic delivery. The liver tightly regulates these pathways transcriptionally via the SREBP1 and SREBP2 (11). Specific to cholesterol metabolism, SREBP2 senses cholesterol within the endoplasmic reticulum and when cholesterol levels are decreased, it is translocated to the nucleus where it activates the transcription of cholesterol metabolism-related genes. SREBP2 transcriptionally regulates all genes required for de novo cholesterol biosynthesis (hydroxymethylglutaryl-CoA synthase 1[*Hmgcs1*], 3-hydroxy-3-methylglutaryl-CoA reductase (*Hmgcr*), and so on), cholesterol esterification (*Acat2*), cholesterol uptake (LDL receptor [*Ldlr*]), and cholesterol feedback inhibition (*Pcsk9, Insig1*, and *Sesn1*) (11, 12). Due to the strong transcriptional regulation of cholesterol metabolism within the liver, it is an ideal biological system to study using genome-wide transcriptomic and proteomic approaches.

In this study, we take advantage of the strong transcriptional regulation of liver cholesterol metabolism by leveraging publicly available mouse transcriptome and proteome datasets to identify unrecognized genes that regulate cholesterol metabolism. By virtue of the liver’s importance in whole-body metabolism and its ease of collection and processing, mouse systems genetics studies commonly perform genome-wide profiling of the liver’s transcriptome and proteome. In previous work, we have shown the utility of module-based coexpression networks within the liver to identify a module of genes, significantly enriched for the gene ontology (GO) term, cholesterol biosynthetic process (12). Here, we applied module-based weighted gene coexpression analysis (WGCNA), to a comprehensive catalog of 35 genome-wide mouse liver expression datasets obtained from mouse genetic reference populations and F2 mouse crosses (13, 14). To our knowledge, this comprehensive catalog of 35 mouse datasets (encompassing more than 3,800 mice), includes nearly all genome-wide mouse liver expression datasets performed in the past twenty years.

By leveraging the 35 mouse liver expression datasets, we identified four genes, retinol dehydrogenase 11 (*Rdh11*), ethylmalonyl-CoA decarboxylase 1 (*Echdc1*), aldolase C (*Aldoc*), and polyamine oxidase (*Paox*) that were replicated more than ten times across the individually identified cholesterol modules. As these genes do not have a previously described role in cholesterol metabolism, we functionally validated that the top three of these genes (*Rdh11, Echdc1, and Aldoc*) can modulate cholesterol metabolism. For the glycolytic gene, *Aldoc*, we performed in-depth biochemical studies and demonstrate that *Aldoc* regulates cholesterol metabolism through the regulation of de novo cholesterol biosynthesis. In physiological studies using multiple mouse models of *Aldoc*, we demonstrate a role for *Aldoc* in regulating liver and whole-body lipid metabolism. Finally, cross-species integration with human lipid GWAS data identified that *Aldoc* is located within a genome-wide significant locus for TC and LDL-C on chromosome 17 and coupled with our validation data indicates *Aldoc* is a causal gene within this locus. Taken together, our work provides a framework to discover genes involved in cholesterol metabolism and a deeper understanding of genes that contribute to dyslipidemia and other lipid-related disorders.

## Materials and methods

### WGCNA of mouse liver datasets

Liver expression and proteome data were obtained from mouse genetic reference populations including the Hybrid Mouse Diversity Panel, diversity outbred, and BXD population of mice as well as F2 crosses of two mouse strains (15, 16, 17, 18, 19, 20, 21, 22, 23, 24, 25, 26, 27, 28, 29, 30). Information on the mouse liver datasets used in this study are available in Table 1 and Supplemental Table S1. Network construction was performed using the WGCNA package in R (13, 14). Network analysis was performed using normalized data on all quantified transcripts or proteins as described previously (12).

**Table 1:** A Comprehensive Catalog of Mouse Liver Expression Datasets

Dataset	Mouse Population	Sex	No. of Mice	Genes in Module	Enrichment *P*-value (Cholesterol Biosynthetic Process)
1	Hybrid mouse diversity panel	Male	288	787	3.9 × 10^−7^
2	Hybrid mouse diversity panel	Male	227	165	1.9 × 10^−18^
3	Hybrid mouse diversity panel	Female	206	372	7.0 × 10^−15^
4	Hybrid mouse diversity panel	Male	78	85	5.2 × 10^−19^
5	Diversity outbred	Male	48	108	6.0 × 10^−26^
6	Diversity outbred	Female	50	68	2.8 × 10^−25^
7	Diversity outbred	Male	46	206	9.5 × 10^−18^
8	Diversity outbred	Female	48	642	7.0 × 10^−14^
9	Diversity outbred	Male	48	61	5.3 × 10^−13^
10	Diversity outbred	Female	50	77	8.8 × 10^−23^
11	Diversity outbred	Male	46	57	7.5 × 10^−16^
12	Diversity outbred	Female	48	35	5.8 × 10^−27^
13	Hybrid mouse diversity population	Male	115	189	3.7 × 10^−35^
14	F2: C57BL/6J-ApoE^−/−^ and C3H/HeJ-ApoE^−/−^	Female	142	200	8.3 × 10^−19^
15	F2: C57BL/6J-ApoE^−/−^ and C3H/HeJ-ApoE^−/−^	Male	142	50	4.9 × 10^−30^
16	Hybrid mouse diversity population	Female	96	138	1.4 × 10^−18^
17	Hybrid mouse diversity population	Male	101	73	4.6 × 10^−22^
18	Hybrid mouse diversity population	Male	295	13	1.2 × 10^−9^
19	F2: CAST/EiJ and C57BL/6J	Male	173	41	5.5 × 10^−3^
20	F2: CAST/EiJ and C57BL/6J	Female	249	12	2.1 × 10^−2^
21	Hybrid mouse diversity population	Male	97	88	2.3 × 10^−4^
22	Hybrid mouse diversity population	Male	99	69	3.3 × 10^−41^
23	Hybrid mouse diversity population	Male	96	146	3.24 × 10^−29^
24	F2: C57BL/6J and DBA/2J	Female	111	44	2.1 × 10^−4^
25	F2: C57BL/6J Lep^ob/ob^ and BTBR-T+ Itpr3tf/J Lep^ob/ob^	Female	254	35	3.0 × 10^−24^
26	F2: C57BL/6J Lep^ob/ob^ and BTBR-T+ Itpr3tf/J Lep^ob/ob^	Male	220	28	3.8 × 10^−28^
27	F2: C57BL/6J and C3H/HeJ	Female	77	132	1.5 × 10^−10^
28	F2: C57BL/6J and C3H/HeJ	Male	82	18	8.6 × 10^−15^
29	BXD family	Male	25	36	1.2 × 10^−13^
30	BXD family	Male	40	186	7.1 × 10^−26^
31	BXD family	Male	40	300	2.7 × 10^−17^
32	BXD family	Male	53	14	3.2 × 10^−2^
33	BXD family	Male	36	33	3.0 × 10^−3^
34	BXD family	Male	41	32	5.6 × 10^−15^
35	BXD family	Female	41	58	9.3 × 10^−17^

BXD, C57BL/6J X DBA/2J.

### Human lipid GWAS datasets

Associations of ALDOC with human cholesterol traits were visualized using summary statistics uploaded to LocusZoom from the multiancestry Global Lipid Genetic Consortium GWAS (5, 31).

### Cell culture experiments

Alpha mouse liver 12 (AML12) cells were maintained in F12/DMEM 50/50 medium (Corning, 10-092-CV) and 10% FBS (Corning, 35-010-CV) with supplementation with insulin-transferrin-selenium (Corning, 354350) for maintenance. For siRNA knockdown experiments, 2.5 × 10^5^ AML12 cells were plated 24 h before siRNA transfection. Cells were transfected with 50 nM scrambled (control) or targeted SiRNAs together with jetOPTIMUS transfection reagent and buffer (Polyplus-transfection, 117-07) in F12/DMEM 50/50 medium supplemented with 10% FBS. All siRNA-related experiments were performed 48 h after transfection allowing sufficient gene knockdown. All siRNAs used in AML12 cells were purchased from Qiagen and product numbers for each are listed as follows: aldolase a (Aldoa) (SI0089623), aldolase b Aldob (SI00236033), Aldoc (SI00896259), Rdh11 (SI00219443 and SI02694860) and Control (1027281). Cholesterol depletion was performed using either replacement of 10% FBS media with 5% lipoprotein deficient serum (LPDS) (Alfa Aesar, J65182) alone or in combination with 0.5% methyl-beta cyclodextrin (MBCD) (Alfa Aesar, J66847). Cellular cholesterol quantification was performed using Amplex™ Red Cholesterol Assay Kit (Thermo Fisher Scientific, A12216) and performed per manufacturer instructions. The aldolase (ab196994) activity assays were from Abcam and performed per manufacturer instructions. HEPG2 cells were maintained in EMEM medium supplemented with 10% FBS. For Srebp2 siRNA knockdown experiments, 250,000 HEPG2 cells were plated 24 h before siRNA transfection. Cells were transfected with 50 nM control (Qiagen, 1027281) or targeted Srebp2 SiRNAs (Qiagen, SI00065286, SI00065863, SI00065870, and SI03029481) together with jetOPTIMUS transfection reagent and buffer (Polyplus transfection, catalog #117-07) and 48 h later, RNA was isolated.

### Quantification of glucose and acetate conversion to cholesterol

AML12 cells were cultured as described above. Radiolabeled glucose D-[6–^14^C] and acetic acid [U-^14^C] was obtained from American Radiolabeled Chemicals. 48 h after transfection cells were treated with 0.5% MBCD in 5% LPDS for 4 h after which cells were switched into media containing 5% LPDS and 1 uCi of glucose D-[6–^14^C] or 1 uCi of acetic acid [U-^14^C] for 4 h. After 4 h, cells were washed twice with PBS and collected via trypsinization. Cells were then spun down and resuspended in PBS with 10% of cells saved for protein quantification; 10% of cells were counted for total cellular radioactivity and the other 80% were extracted for lipid content using a 2:1 chloroform to methanol extraction. Lipids were resuspended in 50 μL of EtOH and spotted onto silica thin layer plates (Millipore Sigma, 1.00390.0001). Chromatography was carried out in hexane/diethyl ether/acetic acid (80:20:1). Cholesterol was identified by cochromatography of a cholesterol standard visualized by iodine vapor staining. Samples were scraped from the TLC plates and resuspended in 6 ml of scintillation cocktail and counted on a liquid scintillation analyzer (PerkinElmer, Tri-Carb© 2910 TR).

### Metabolomics

Metabolites were measured using a Thermo Q-Exactive mass spectrometer coupled to a Vanquish Horizon UHPLC. The data were collected on a full scan negative mode. The metabolites identified were based on exact *m/z* and retention times that were determined with chemical standards. Data were collected with Xcalibur 4.0 (www.thermofisher.com/order/catalog/product/OPTON-30965) software and peak integration was performed using MAVEN (http://maven.princeton.edu) (32). Relative metabolite levels were normalized to cellular protein content. After isolation in −80°C 80:20 MeOH:H_2_O, metabolite extracts were dried under nitrogen stream. Samples were resuspended in LC-MS grade water and separated on a 2.1 × 100 mm, 1.7 μM Acquity UPLC BEH C18 Column (Waters). The solvents used were A: 97:3 water:methanol (v:v), 10 mM tributylamine, 9 mM acetate, pH 8.2 and B: 100% methanol. The gradient was 0 min, 95% A; 2.5 min, 95% A; 17 min, 5% A; 21 min, 5% A; and 21.5 min, 95% A. The flow rate was 0.2 ml/min and the column temperature was 30°C.

### Animal experiments

C57BL/6NJ-*Echdc1*^*em1(IMPC)J*^/Mmjax mice were purchased from Jackson laboratories and generated by the KO Mouse Phenotyping Program. *Echdc1*^−/−^ mice were specifically generated by a deletion of exon 3. C57BL/6N-*Aldoc*^*tm1.1(KOMP)Vlcg*/MbpMmucd^ mouse sperm was obtained from the Mutant Mouse Resource and Research Centers. The University of Wisconsin-Madison Biotechnology center performed in vitro fertilization on WT oocytes from C57BL/6N mice to generate Aldoc ^+/−^ mice. *Aldoc* ^−/−^ mice were bred to *Ldlr* ^−/−^ mice (B6.129S7-Ldlr^tm1Her/J^) to generate *Ldlr* ^−/−^ *Aldoc* ^+/+^ and *Ldlr* ^−/−^ *Aldoc* ^−/−^ mice. All experimental mice were generated using heterozygous crosses. *Echdc1* and *Aldoc*-*Ldlr* mice were fed with 2020X chow diet (Teklad). *Aldoc* mice were fed with 8604 chow diet (Teklad). Unless otherwise noted, mice were sacrificed or bled under isoflurane anesthetic after a 4-h morning fast. Blood was collected in EDTA coated tubes (MiniCollect®, 450475). The experimental AAV8-TBG-*Aldoc* (AAV8, adeno-associated virus 8 and thyroxine-binding globulin (TBG)) virus was purchased from Vector Biolabs. The control AAV8-TBG-*Gfp* virus was purchased from Addgene. Mice were injected retroorbitally with 1 x 10^11^ genome copies of AAV8 at 8–10 weeks of age. Mice treated with AAV8-TBG were refed with a purified 70% sucrose diet (Teklad, TD.98090). Cholesterol and lovastatin studies were performed as previously described (12). All experimental procedures were performed with approval from the IACUC at the University of Wisconsin-Madison.

### Quantitative PCR

Total RNA was extracted in QIAzol reagent (Qiagen, 79306) according to manufacturers’ recommendations. Subsequently, 1,000 ng of total RNA was reversed transcribed to complementary DNA by High-Capacity cDNA Reverse Transcription Kit (Thermo Fisher Scientific, 4368813). The quantitative PCR assay was performed using KAPA-SYBR-FAST quantitative PCR master mix kit (Roche, KK4611) in a Roche LightCyler 480 real time PCR machine. The concentration of mRNA targets for each sample were calculated by the Roche LightCycler 480 software (https://lifescience.roche.com/global/en/products/product-category/lightcycler.html) based off of a standard curve and each target mRNA was normalized to the reference gene, *Rpl4*. Primer sequences as follows organized as gene (Species, forward primer, reverse primer):

*Rpl4* (Mouse, AGCAGCCGGGTAGAGAGG, ATGACTCTCCCTTTTCGGAGT),

*Aldoa* (Mouse, TGGGAAGAAGGAGAACCTGA, GACAAGCGAGGCTGTTGG,

*Aldob* (Mouse, GGCTGGTCCCTATTGTTGAG, TAGACAGCAGCCAGGACCTT),

*Aldoc* (Mouse, CCTGGAGAGGACAAAGGGATA, TGCAAGCCCGTTCATCTC),

*Hmgcs1* (Mouse, TCCCCTTTGGCTCTTTCACC, GGGCAACGATTCCCACATCT),

*Hmgcr* (Mouse, CGTGAGGGTCGTCCAATTT, TGAACAAGGACCAAGCCTAAA),

*Abca1* (Mouse, GGTTTGGAGATGGTTATACAATAGTTGT, CCCGGAAACGCAAGTCC)

*Rdh11* (Mouse, GAGGAGCCCGTGTGTATTTAG, GGTATCAGCTAGGTCCAGTTTC),

*Echdc1* (Mouse, GCCATGACACTCCCAATACA, AGAAGGAGAAAGAGAGGGAGAA),

Fdft1 (Mouse, CCAAACAGGACTGGGACAAG, GACGAGAAAGGCCAATTCC),

Mvk (Mouse, ACGAGCTTTCTTGGCCTCTC, TGGGTACCGAGACATCACCT),

Idi1 (Mouse, CGAGCGATTGGATATGCTG, AATGTCTGATCTGACCTAGAACACAG),

Srebp1c (Mouse, CGACTACATCCGCTTCTTGCAG, CCTCCATAGACACATCTGTGCC),

Ldlr (Mouse, CCAATCGACTCACGGGTTCA, CTCACACCAGTTCACCCCTC),

Paox (Mouse, GGAAGATACATCGCCCTTACAG, GACTCCAGCCCAGCAATAAA),

Abca1 (Mouse, GGTTTGGAGATGGTTATACAATAGTTGT, CCCGGAAACGCAAGTCC),

Lpcat3 (Mouse, TAACCGCCCTTTCTGGTTCC, GCACACTCCTTCTGTGACCA),

Fasn (Mouse, TTGGCCCAGAACTCCTGTAG, CTCGCTTGTCGTCTGCCT),

Scd1 (Mouse, TCGCCTACACCAACGGG, GTGTAAGAACTGGAGATCTCTTGGA),

*RPL4* (Human, CAAAAACGATACGCCATCTG, GAACTTCCTCAATACGATGACCTT),

*ALDOC* (Human, GGATCAGAACCCGAGCTGT, GCCACCCTCTTCTCTCAGC),

*HMGCR* (Human, GCCACCCTCTTCTCTCAGC, GCTGCCAAATTGGACGAC),

RDH11 (Human, GCGCCCCAAATCAGGAAAAT, TGGCTGTCTCCTTCCCGATA),

ECHDC1 (Human, GGGTGGAGGAGCAGAATTTAC, CCATCTCTTTGTGGACGAATCT),

PAOX (Human, ACCTTTCCAGTGTCGGTAGA, GGTGTCCAAATGTTCCCTAAGA).

### Plasma protein, total cholesterol, triglycerides, and glucose analysis

Protein content was quantified using a Pierce BCA protein assay kit (Thermo Fisher Scientific, 23225). TC (C7510), triglycerides (T7532), and glucose (G7521) were analyzed with indicated colorimetric kits (Pointe Scientific). Liver lipids were extracted using the Folch method (33). Briefly mouse liver tissue (∼100 mg) was homogenized in methanol after which 2X volumes of chloroform were added. Homogenates were incubated at 4°C before liver tissue was filtered out of the solvent, and the solvent was treated with 0.2X volumes of 0.43% MgCl_2_. Samples were allowed to settle into aqueous and organic phases before the aqueous phase was removed, and the organic phase was dried down and resuspended in 100% ethanol, then assayed using the listed Pointe Scientific kits.

### Fast protein liquid chromatography separation of mouse plasma

Size-exclusion chromatography was performed on an AKTA fast-protein liquid chromatography (FPLC) (Amersham pharmacia biotech). Equivalent volumes of plasma from each group of mice were pooled, totaling 500 μl (*Echdc1* and *Aldoc*) or 300 μl of plasma (*Aldoc*-*Ldlr*). Plasma was diluted in PBS so total sample volume equaled 1,000 μl and was applied to a Superose 6 followed in tandem with a Superdex 200 column and separated into lipoprotein classes in 10 mM PBS, pH 7.4, containing 0.02% sodium azide and collected into 48, 0.5 ml fractions. Fractions were then analyzed for protein, triglycerides, and cholesterol content as indicated above. Fractions were analyzed for apolipoprotein B (ApoB) abundance as follows. FPLC fractions were incubated at 95°C for 10 min in 2X laemmli buffer (Bio-Rad) before being subjected to SDS-Page electrophoresis on 4%–20% Mini-PROTEAN TGX gels (Bio-Rad). Gels were then transferred to 0.45-mm nitrocellulose membranes at 350 mA for 1.5 h. Membranes were blocked in 5% non-fat dried milk tris buffered saline-tween-20 overnight at 4°C before being incubated with Anti-ApoB antibody (1:1,000, ab20737, Abcam) for 2 h at room temperature before incubation with goat anti-rabbit secondary antibody (1:20,000, 926–32211, LICOR) for 1 h at room temperature.

### Glucose tolerance test

Glucose (Thermo Fisher Scientific, 492-62-6) tolerance tests were performed after a 24 h fast with 1 g/kg administered via oral gavage.

### Statistical analysis

Statistical analysis of in vitro and in vivo validation data was performed using student’s two-tailed t tests or one-way ANOVA with Tukey’s post hoc test when appropriate. Statistical test used is indicated within figure legend. Statistical significance was defined as follows. ∗*P* < 0.05, ∗∗*P* < 0.01, ∗∗∗*P* < 0.001 and ∗∗∗∗*P* < 0.0001. Analysis was performed in Graphpad Prism (https://www.graphpad.com/).

## Results

### Module-based coexpression network construction and gene prioritization

In mouse systems genetics studies, it is commonplace to use the liver for assessment of genome-wide transcript (transcriptomics) and protein (proteomics) abundance. Given the tremendous wealth of mouse liver transcriptomics and proteomics data publicly available, we obtained 35 distinct datasets from mouse systems genetics studies. These 35 liver datasets encompass 3,808 male and female mice in total and include datasets from the following genetic reference populations; Hybrid Mouse Diversity Panel (11 datasets) (15, 16, 17, 20, 22, 23, 29), diversity outbred (8 datasets) (18), and C57BL/6J X DBA/2J (BXD family) (7 datasets) (27, 28, 30). We also obtained nine datasets from F2 crosses between the following mouse strains; C57BL/6J X C3H/HeJ (2 datasets) (26), B6.Cg-ApoE^−/−^ X C3H.Cg-ApoE^−/−^ (2 datasets) (19), CAST/EiJ X C57BL/6J (2 datasets) (21), B6.Cg-Lep^*ob/ob*^/J X BTBR.Cg-Lep^*ob/ob*^/WiscJ (2 datasets) (25), and C57BL/6J X DBA/2J (1 dataset) (24). Represented across these liver datasets are both male and female mice, multiple dietary and chemical interventions, as well as studies with mutations for key metabolic genes (apolipoprotein E and Leptin) (Table 1 and Supplemental Table S1). Taken together, this collection of 35 genome-wide transcriptome and proteome liver datasets represents a comprehensive catalog of nearly all liver data available from mouse systems genetics studies.

**Fig. 1:**
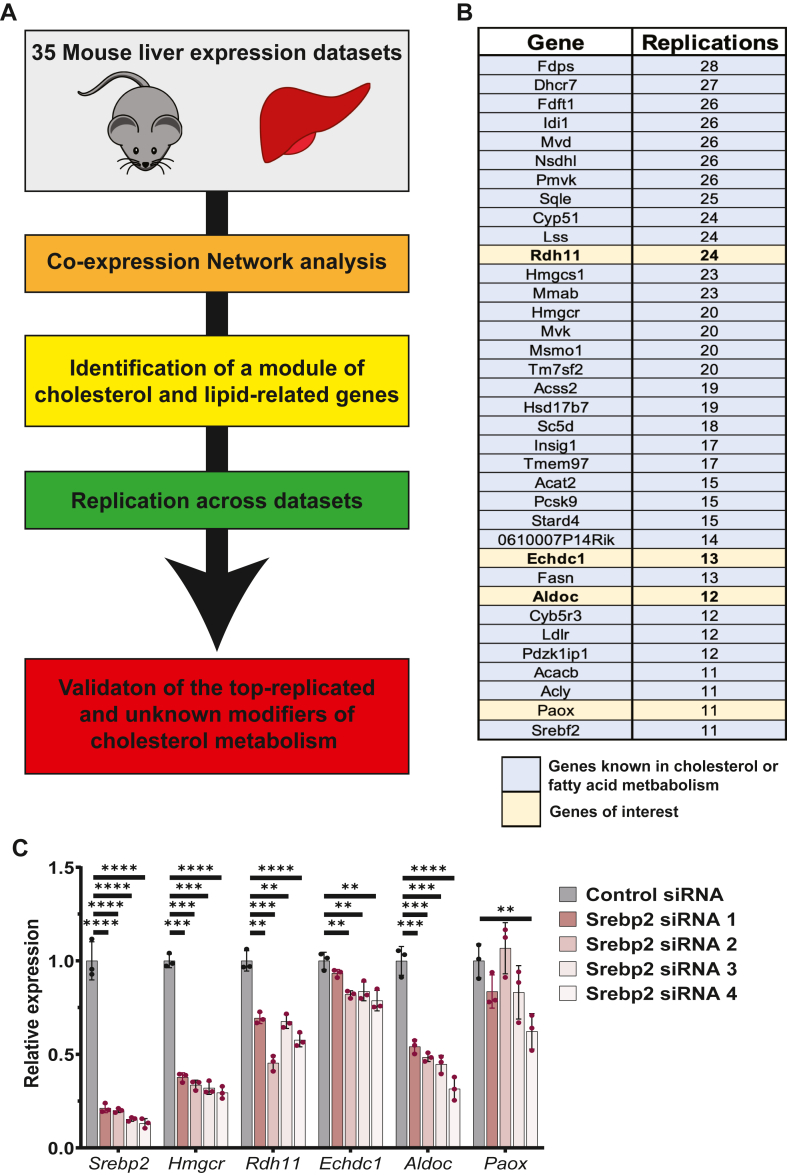
Identification of cholesterol-related genes using module-based coexpression analysis of 35 mouse liver genome-wide expression datasets. A: Schematic representation of framework for systematic analysis of 35 mouse liver genome-wide expression datasets for the identification of new genes involved in cholesterol metabolism. B: Identification of 36 genes that were replicated more than 10 times within a module of genes, enriched for the GO term “cholesterol biosynthetic process” across the 35-mouse liver genome-wide expression datasets. Highlighted in *blue* are genes with known role in cholesterol and fatty acid metabolism. Highlighted in *tan* are prioritized genes of interest with no known role in cholesterol metabolism. C: Relative mRNA expression of *Srebp2, Hmgcr, Rdh11, Echdc1, Aldoc*, and *Paox* in human HEPG2 cells after transfection with either control (Scrambled) or 1 of 4 *Srebp2*-directed siRNAs. Statistical differences determined with an unpaired two-tailed *t* test, denoted by ∗∗*P* < 0.01, ∗∗∗*P* < 0.001, and ∗∗∗∗*P* < 0.0001. Aldoc, aldolase C; Echdc1, ethylmalonyl-CoA decarboxylase 1; GO, gene ontology; Paox, polyamine oxidase; Rdh11, retinol dehydrogenase 11.

In previous work, we demonstrated that coexpression network analysis of liver transcriptomic and proteomic data can be used to identify a conserved module of genes highly enriched for the GO term cholesterol biosynthetic process (12). Building upon this concept, we wanted to leverage this comprehensive catalog of 3,808 mouse livers and develop a systematic pipeline to quickly move from module-based network analysis to gene identification and functional validation of genes not known to be involved in cholesterol metabolism. This systematic pipeline involves *1*) module-based network construction *2*) identifying the module of genes significantly enriched for the GO term “cholesterol biosynthetic process”, *3*) ranking of genes based on replication across the 35 datasets, and *4*) functional validation of top-ranking unknown genes (Fig. 1A).

For each of the 35 liver datasets, we performed WGCNA to cluster gene transcript and protein abundance data into modules of coexpressed genes (14). In all 35 datasets, we were able to identify a module of genes significantly enriched for the GO term “cholesterol biosynthetic process” (enrichment *P* values range from 3.2 × 10^−2^ to 3.3 × 10^−41^) (Table 1). As this module is highly enriched for the GO term “cholesterol biosynthetic process”, we refer to the module as the “cholesterol” module. This cholesterol module ranges in size from 12 to 823 genes, depending on the dataset, and altogether includes 3,085 unique genes across all 35 datasets (Table 1 and Supplemental Table S2). Within the cholesterol modules, many genes are well-known to regulate diverse aspects of cholesterol metabolism while others do not have a defined role in cholesterol metabolism. It should be noted that the cholesterol module is also significantly enriched for the GO term “fatty acid biosynthetic process” and contains genes known to be involved in this process.

To systematically prioritize all the 3,085 unique genes contained within all the cholesterol modules, we ranked the genes based on replication across the 35 datasets and identified 36 genes that replicated more than ten times (Fig. 1B and Supplemental Table S3). Out of these 36 genes, 32 have described roles in cholesterol or fatty acid metabolism (Supplemental Fig. S1). Nineteen belong to the cholesterol biosynthetic pathway starting from acetyl-CoA and ending with the synthesis of cholesterol. Nine participate in the transcriptional regulation of cholesterol genes (*Srebf2* and *Insig1*), the uptake of cholesterol (*Ldlr* and *Pcsk9*), or contribute to the transport or regulation of cholesterol metabolism (metabolism of cobalamin associated B (*Mmab*), transmembrane protein 97 (*Tmem97*), *Stard4*, cytochrome B5 reductase 3 (*Cyb5r3*), and *Pdzk1ip1*). Four participate in fatty acid biosynthesis (*Fasn* and acetyl-CoA carboxylase beta [*Acacb*]) and the production of cytosolic acetyl-CoA (*Acly* and *Acss2*), which can be utilized in the cholesterol or fatty acid biosynthetic pathways. Collectively, from the replication analysis we were able to identify four genes (*Rdh11, Echdc1, Aldoc*, and *Paox*) that did not have described roles in cholesterol or fatty acid metabolism.

As many of the top 36 replicated genes are known transcriptional targets of SREBP2, we tested transcriptional regulation of *Rdh11*, *Echdc1*, *Aldoc*, and *Paox* by SREBP2. Targeting *Srebp2* with four distinct siRNAs in human HEPG2 hepatocyte cells resulted in greater than an 80 percent knockdown of *Srebp2*. *Srebp2* knockdown led to a significant reduction of the known target gene *Hmgcr* and a significant reduction in *Rdh11* and *Aldoc* (Fig. 1C). For *Echdc1*, three of the four siRNAs targeting *Srebp2* led to a significant reduction in *Echdc1* expression. Only one siRNA targeting *Srebp2* resulted in a significant reduction in *Paox* expression (Fig. 1C). Furthermore, *Paox* expression was not regulated by cholesterol depletion in AML12 hepatocyte cells depleted of cholesterol or by treatment with LDL-C (Supplemental Fig. S2). These data corroborate previous publications showing hepatic *Rdh11* and *Aldoc* are regulated by SREBP2 (34, 35). Furthermore, transcriptional regulation by SREBP2 of *Echdc1* is supported by chromatin immunoprecipitation sequencing studies for SREBP2, which identified *Echdc1* as one of the 1,500 genes with SREBP2 binding (36). Therefore, based on SREBP2 regulation and replication we prioritized *Rdh11*, *Echdc1*, and *Aldoc* to follow-up and understand their role in cholesterol homeostasis.

### Functional validation of *Rdh11* as a cholesterol-related gene

**Fig. 2:**
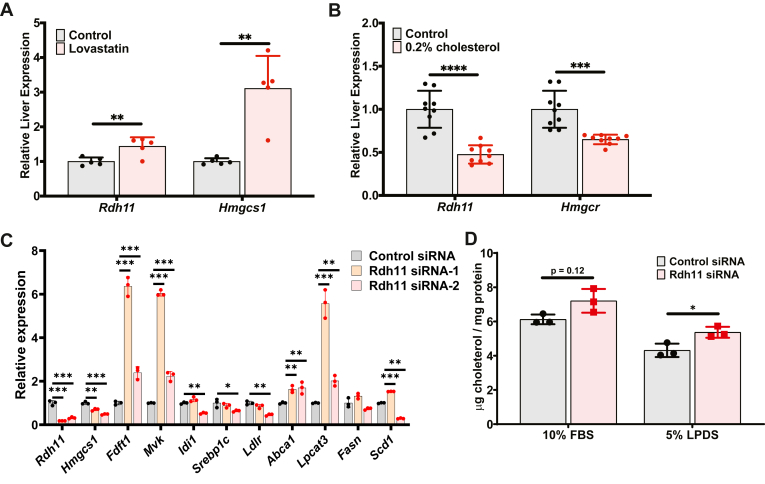
Validation of Rdh11 as a cholesterol-related gene. A: Relative liver mRNA expression of *Rdh11* and *Hmgcs1* of mice fed a control diet or diet containing 0.02% lovastatin. B: Relative liver mRNA expression of *Rdh11* and *Hmgcr* fed either a control diet or diet containing 0.2% cholesterol. C: Relative mRNA expression of *Rdh11*, *Hmgcs1*, *Fdft1*, *Mvk, Idi1, Srebp1c, Ldlr, Abca1, Lpcat3, Fasn*, and *Scd1* in mouse AML12 hepatocyte cells transfected with either control (Scrambled) or 1 of 2 *Rdh11*-directed siRNAs and incubated with 5% LPDS for 16 h. D: Analysis of cellular cholesterol concentration in mouse AML12 hepatocyte cells after being transfected with either control (Scrambled) or Rdh11 siRNA and incubated with either 10% FBS or 5% LPDS. Data presented as mean ± SD. Statistical differences were determined by unpaired two-tailed *t* test denoted by ∗*P* < 0.05, ∗∗*P* < 0.01, ∗∗∗*P* < 0.001, and ∗∗∗∗*P* < 0.0001. AML12, alpha mouse liver 12; Hmgcs1, hydroxymethylglutaryl-CoA synthase 1; Ldlr, low-density lipoprotein receptor; LPDS, lipoprotein deficient serum; Rdh11, retinol dehydrogenase 11.

To functionally validate *Rdh11*, we first tested the transcriptional regulation of hepatic *Rdh11* in vivo in response to perturbed cholesterol homeostasis. When mice are fed a diet supplemented with lovastatin (to inhibit hepatic cholesterol biosynthesis) for one week, the relative expressions of liver *Rdh11* and the SREBP2 target gene, *Hmgcs1* are significantly increased compared to control mice (Fig. 2A). On the contrary, when mice are fed a diet containing 0.2% cholesterol or a control diet with no cholesterol added, the relative expressions of *Rdh11* and the SREBP2 target gene, *Hmgcr* (HMG-CoA reductase) are significantly reduced in mice fed a 0.2% cholesterol diet (Fig. 2B). These data are consistent with our studies demonstrating transcriptional regulation of *Rdh11* by SREBP2 (Fig. 1C) and prior studies indicating hepatic *Rdh11* is regulated by SREBP2 in mice (34, 35).

To investigate the functional role of *Rdh11* in cholesterol metabolism, we performed targeted studies of *Rdh11* in AML12 hepatocyte. *Rdh11* knockdown with two separate siRNAs led to a strong suppression of *Rdh11* expression in each case (Fig. 2C). Under 5% LPDS conditions, *Rdh11* knockdown resulted in significantly decreased expression of the SREBP2 target genes, *Hmgcs1, Idi1*, and *Ldlr*. Meanwhile the SREBP2 target genes *Fdft1* and *Mvk*, were significantly increased (Fig. 2C). Expression of the liver X receptor target genes, *Abca1* and *Lpcat3* were significantly increased (Fig. 2C). We also observed a significant decrease in *Srebp1c* expression with one siRNA, no change in *Fasn* expression, and a significant increase in *Scd1* expression with one siRNA (Fig. 2C). As a result of the significant alterations in SREBP2 and liver X receptor target genes, we isolated lipids from AML12 cells after *Rdh11* knockdown and quantified total cellular cholesterol content within cells incubated in 10% FBS or LPDS for 24 h, to promote cholesterol biosynthesis. After *Rdh11* knockdown, the concentration of cellular cholesterol was significantly increased in cells incubated in 5% LPDS (Fig. 2D). Taken together, these data validate our network-based identification and prioritization of *Rdh11* as a cholesterol-related gene. Our functional validation studies demonstrate that *Rdh11* can regulate cellular cholesterol levels under conditions of elevated cholesterol biosynthesis.

### Functional Validation of *Echdc1* as a cholesterol-related gene

**Fig. 3:**
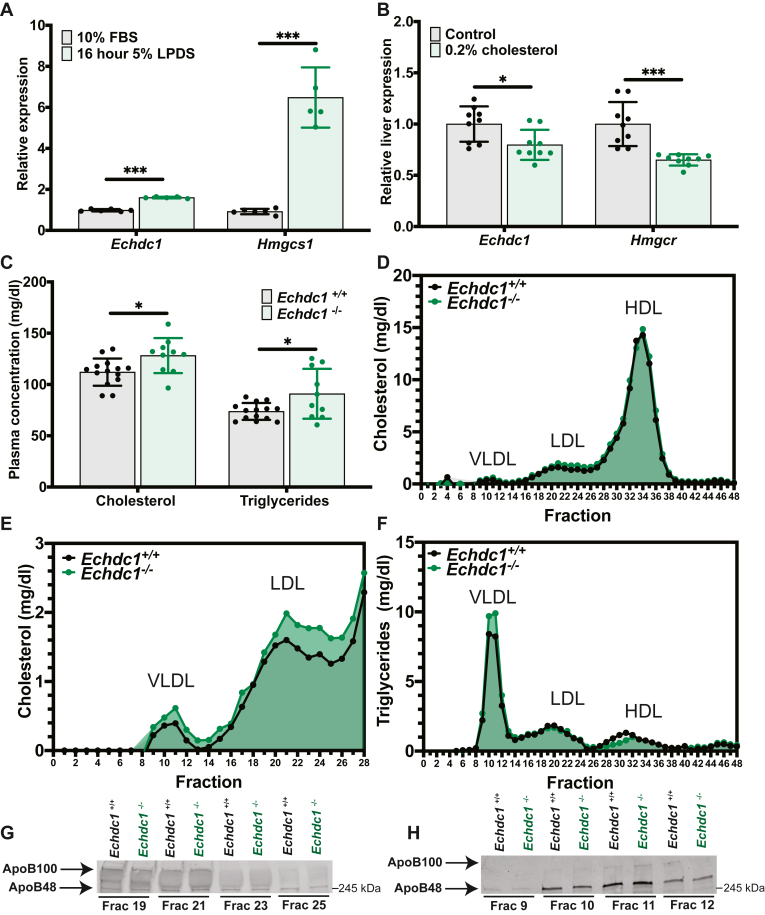
Validation of Echdc1 as a cholesterol-related gene. A: Relative mRNA expression of *Echdc1* and *Hmgcs1* in mouse AML12 hepatocyte cells after incubation with 10% FBS or 5% LPDS for 16 h. B: Relative liver mRNA expression of *Echdc1* and *Hmgcr* from mice fed either a control diet or diet containing 0.2% cholesterol. C: Plasma concentration of cholesterol and triglycerides in male (8-week-old) Echdc1^+/+^ and Echdc1^−/−^ mice fasted for four hours. Plasma fractionation by FPLC from male (8-week-old) Echdc1^+/+^ and Echdc1^−/−^ mice displaying (D) cholesterol concentration in all fractions, (E) cholesterol concentration in non-HDL fractions, and (F) triglyceride concentration in all fractions. Western blot analysis for ApoB48 and ApoB100 in indicated fractions for (G) LDL-associated ApoB and (H) VLDL-associated ApoB. Data presented as mean ± SD. Statistical differences were determined with an unpaired two-tailed *t* test denoted by ∗*P* < 0.05, ∗∗*P* < 0.01, ∗∗∗*P* < 0.001, and ∗∗∗∗*P* < 0.0001. AML12, alpha mouse liver 12; ApoB, apolipoprotein B; Echdc1, ethylmalonyl-CoA decarboxylase 1; FPLC, fast-protein liquid chromatography; Hmgcs1, hydroxymethylglutaryl-CoA synthase 1; LPDS, lipoprotein deficient serum.

To functionally validate *Echdc1* (Ethylmalonyl-CoA Decarboxylase 1) as a cholesterol-related gene, we first tested the transcriptional regulation of *Echdc1* by cholesterol in vitro and in vivo. In AML12 hepatocyte cells treated with 5% LPDS, the relative expression of *Echdc1* and the SREBP2 target gene, *Hmgcs1* was significantly increased compared to control-treated cells maintained in FBS (Fig. 3A). In mice, feeding a diet containing 0.2% cholesterol versus a control diet with no cholesterol resulted in a significant decrease in the relative hepatic expression of *Echdc1* and the SREBP2 target gene, *Hmgcr* (Fig. 3B). These data demonstrate that hepatic *Echdc1* is transcriptionally regulated by cholesterol, which is consistent with transcriptional regulation by SREBP2 (Fig. 1C) and other studies showing binding of SREBP2 to the Echdc1 gene promoter (36).

To functionally validate the role of *Echdc1* and test if deletion of *Echdc1* would modulate cholesterol metabolism in mice, we obtained a whole-body *Echdc1* KO mouse (*Echdc1*^−/−^). Measuring plasma levels of cholesterol and triglycerides in male mice fed a standard rodent chow diet revealed that *Echdc1*^−/−^ mice have a significant increase in both plasma TC and triglycerides levels compared to *Echdc1*^+/+^ littermate control mice (Fig. 3C). To further characterize the effect of *Echdc1* on plasma cholesterol and triglycerides, we utilized FPLC to separate very low-density lipoprotein cholesterol (VLDL-C), LDL-C, and HDL-C (Fig. 3D). FPLC fractionation of plasma revealed that *Echdc1*^−/−^ mice have increased VLDL-C and LDL-C as well as increased triglyceride concentration within VLDL fractions (Fig. 3E, F). This increase in LDL-C and VLDL-triglycerides is not the result of an increase in the abundance of LDL or VLDL particles as measured by apolipoprotein B-100 (ApoB100) and apolipoprotein B-48 (ApoB48) protein abundance in relevant fractions (Fig. 3G, H). Increases in plasma levels of cholesterol were independent of hepatic cholesterol and triglyceride levels or hepatic expression of *Srebp2*, *Hmgcs1*, *Hmgcr*, and *Fdft1* (Supplemental Fig. S3A–C). Taken together, our functional validation studies in mice demonstrate that *Echdc1* modulates plasma VLDL-C, LDL-C, and VLDL-triglyceride levels and establishes *Echdc1* as a previously unrecognized gene involved in regulating plasma cholesterol and triglycerides.

### Functional validation of *Aldoc* as a cholesterol-related gene

**Fig. 4:**
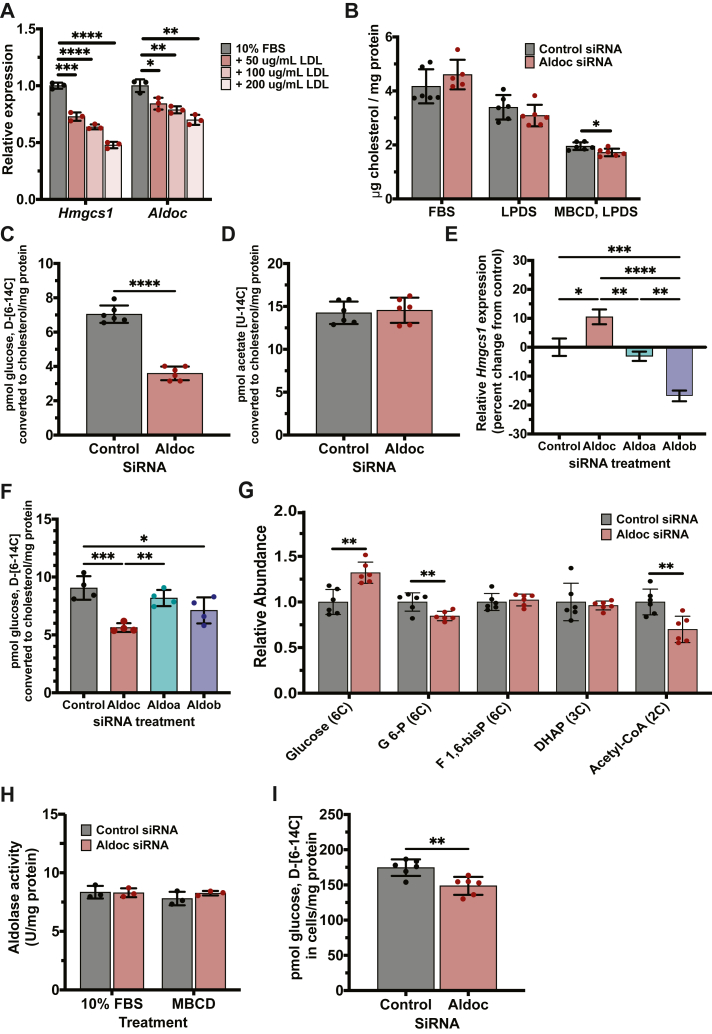
Validation of Aldoc as a cholesterol-related gene. A: Relative mRNA expression of *Hmgcs1* and *Aldoc* in mouse AML12 hepatocyte cells incubated with 10% FBS and 3 different concentrations of human LDL-C. B: Analysis of cellular cholesterol concentration in mouse AML12 hepatocyte cells after being transfected with either control (Scrambled) or *Aldoc* siRNA and incubated with 0% FBS, 5% LPDS for 16 h, or 4 h with 0.5% MBCD/5% LPDS followed by 4 h with 5% LPDS. Analysis of glucose or acetate converted into cholesterol in mouse AML12 hepatocyte cells transfected with control (Scrambled) or *Aldoc*-directed siRNA and treated for 4 h with 0.5% MBCD/5% LPDS and 4 h with 5% LPDS with (C) 1 μCi of glucose D-[6–14C] or (D) 1 μCi of acetic acid [U-14C]. E: Relative mRNA expression of *Hmgcs1* in mouse AML12 hepatocytes represented as percent change from control after transfection with control (Scrambled), *Aldoc, Aldoa*, or *Aldob*-directed siRNAs after incubation with 0.5% MBCD/5% LPDS for 4 h. F: Analysis of glucose converted into cholesterol in mouse AML12 hepatocyte cells transfected with control (Scrambled), *Aldoc, Aldoa*, or *Aldob*-directed siRNAs and treated for 4 h with 0.5% MBCD/5% LPDS and 4 h with 5% LPDS with μCi of glucose D-[6–14C]. G: Relative abundance of [U-13C] labeled polar metabolites in mouse AML12 cells transfected with control (Scrambled) or *Aldoc*-directed siRNA and treated with 0.5% MBCD/5% LPDS for four hours followed by treatment with 5% LPDS and isotopically labeled ^13^C glucose for four hours. H: Aldolase activity in mouse AML12 hepatocyte cells transfected with control (Scrambled) or *Aldoc*-directed siRNA and treated with either 10% FBS or 0.5% MBCD/5% LPDS for four hours. I: Total intracellular ^14^C quantification in mouse AML12 hepatocyte cells transfected with control (Scrambled) or *Aldoc*-directed siRNA and treated with 0.5% MBCD/5% LPDS for four hours followed by treatment with 5% LPDS and 1 μCi of glucose D-[6–14C] for four hours. Data presented as mean ± SD. Statistical differences in A, B, and F were determined by one-way ANOVA with Tukey’s post hoc test. Statistical differences in C to E were determined with an unpaired two-tailed *t* test, both denoted by ∗*P* < 0.05, ∗∗*P* < 0.01, ∗∗∗*P* < 0.001, and ∗∗∗∗*P* < 0.0001. Aldoc, aldolase C; AML12, alpha mouse liver 12; Hmgcs1, hydroxymethylglutaryl-CoA synthase 1; LDL-C, low density lipoprotein cholesterol; LPDS, lipoprotein deficient serum, MBCD, methyl-beta cyclodextrin.

We prioritized *Aldoc* based on being replicated 12 times across the 35 datasets. Previously, we identified *Aldoc* within a mouse liver cholesterol module and found within a GWAS locus associated with LDL-cholesterol levels in humans. We also demonstrated that *Aldoc* knockdown in AML12 hepatocytes leads to an increase in the expression of the SREBP2 target gene, *Hmgcs1* (12). To expand on these results, we first tested the transcriptional regulation of *Aldoc* by cholesterol in vitro. Treatment of AML12 hepatocytes with three concentrations of LDL-cholesterol (50 μg/ml, 100 μg/ml, and 200 μg/ml) resulted in a significant step-down reduction in the relative expression of *Aldoc*, as well as the SREBP2 target gene, *Hmgcs1* (Fig. 4A). These data are consistent with our SREBP2 knockdown results (Fig. 1C) and a previous publication using transgenic overexpression in mice of *Srebp2* along with Srebp cleavage activating protein (*Scap*) deletion, demonstrating *Aldoc* is a transcriptional target of SREBP2 (34).

Next, we tested if *Aldoc* would directly alter cellular cholesterol levels within cells using three separate cell culture conditions; 10% FBS, 5% LPDS, and depletion of cellular cholesterol with 0.5% MBCD. *Aldoc* siRNA-mediated knockdown in AML12 hepatocytes followed by treatment with 10% FBS or 5% LPDS did not alter cellular cholesterol levels. However, in cells depleted of cholesterol with MBCD for 4 h, there was a significant decrease in the amount of cellular cholesterol (Fig. 4B). Cholesterol depletion with MBCD strongly induces de novo cholesterol biosynthesis, and these data suggest that *Aldoc* regulates cellular cholesterol levels when de novo cholesterol biosynthesis is upregulated.

Canonically, *Aldoc* performs the fourth step of glycolysis, the reversible cleavage of fructose 1,6-bisphosphate (F1,6-bisP) into dihydroxyacetone phosphate (DHAP) and glyceraldehyde 3-phosphate and is therefore involved in the conversion of glucose to pyruvate. Pyruvate then enters the mitochondria and is metabolized to acetyl-CoA. Mitochondrial acetyl-CoA is condensed with oxaloacetate to form citrate which can then be exported out of the mitochondria and converted back to acetyl-CoA in the cytosol, where it is utilized for de novo cholesterol biosynthesis. To test if *Aldoc* would specifically alter the conversion of glucose to cholesterol, we used radiolabeled glucose [6–^14^C] and traced its conversion into cholesterol in AML12 cells after MBCD cholesterol depletion. We found *Aldoc* knockdown causes a significant decrease in the amount of glucose converted to cholesterol (Fig. 4C). Next, we tested the ability of *Aldoc* to regulate the conversion of acetyl-CoA to cholesterol using radiolabeled acetic acid [U-^14^C]. Under the same cholesterol-depleted condition used previously, *Aldoc* knockdown did not affect the conversion of acetate to cholesterol (Fig. 4D). These data demonstrate that *Aldoc* impacts de novo cholesterol biosynthesis upstream of the mevalonate pathway.

In mammals, there are three distinct aldolase isozymes, *Aldoa*, *Aldob*, and *Aldoc*. Historically, the three aldolases are recognized based on their tissue of highest expression, with *Aldoa* known as the muscle aldolase, *Aldob* known as the liver aldolase, and *Aldoc* known as the brain aldolase (37). These aldolase isozymes, while closely related, are each unique based on sequence homology, catalytic activities, tissue expression patterns, and genomic location (38). We next tested the isozyme-specific role of *Aldoc* in the regulation of cholesterol metabolism. Using *Hmgcs1* as a readout of SREBP2 activity and cellular cholesterol status, we found that after cholesterol depletion, only *Aldoc* knockdown leads to an increase in the expression of *Hmgcs1*, while *Aldoa* knockdown has no effect, and *Aldob* knockdown leads to a decrease in the expression of *Hmgcs1* (Fig. 4E). Complementary studies using labeled glucose [6–^14^C] demonstrate that *Aldoc* knockdown leads to a significant reduction in glucose conversion to cholesterol while *Aldoa* knockdown had no effect and *Aldob* knockdown led to an intermediate effect that may be related to a decrease in the expression of cholesterol biosynthesis enzymes (Fig. 4F). These data provide strong evidence that *Aldoc* plays an isozyme-specific role in the regulation of de novo cholesterol biosynthesis.

To further understand the role of *Aldoc* in regulating the conversion of glucose to cholesterol, we performed metabolomics analysis under cholesterol-depleted conditions in AML12 cells. In *Aldoc* knockdown cells, there is a significant increase in the ^13^C-labeled abundance of intracellular glucose and a decrease in glucose-6-phosphate (G-6-P) and acetyl-CoA, but there is no difference in the levels of the aldolase substrate and products (F1,6-bisP and DHAP) (Fig. 4G). Consistently, we found that *Aldoc* siRNA knock down has no effect on total aldolase activity in either cholesterol depleted or 10% FBS conditions (Fig. 4H). These results suggest that *Aldoc* is likely to regulate glucose-derived acetyl-CoA production and cholesterol synthesis via mechanism beyond its direct enzymatic reaction. In complimentary studies, we tested total glucose uptake and utilization by measuring total ^14^C abundance in AML12 cells after cholesterol depletion and incubation with ^14^C glucose. In *Aldoc* knockdown cells there was a significantly reduced intracellular ^14^C abundance compared to control cells (Fig. 4I). Collectively, our analyses demonstrate that *Aldoc* is transcriptionally regulated by SREBP2 and plays an isozyme-specific role in the utilization of glucose for de novo cholesterol biosynthesis.

### *Aldoc* regulates plasma cholesterol levels in vivo

**Fig. 5:**
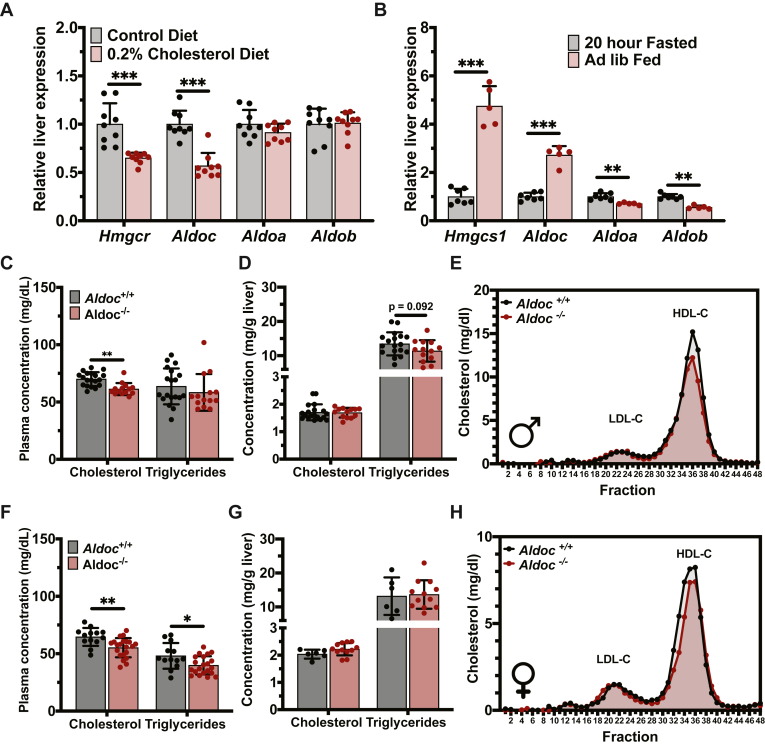
Genetic loss of Aldoc reduces plasma cholesterol in mice. A: Relative liver mRNA expression of *Hmgcr*, *Aldoc*, *Aldoa*, and *Aldob* in mice fed a control diet or diet containing 0.2% cholesterol. B: Relative liver mRNA expression of *Hmgcs1*, *Aldoc*, *Aldoa*, and *Aldob* in the ad-lib fed mice or 20-h fasted mice. (C) Plasma and (D) liver concentrations of cholesterol and triglycerides in male 16-week-old Aldoc^−/−^ and Aldoc^+/+^ littermate control mice fasted for four hours. E: Analysis of cholesterol-containing lipoproteins by FPLC fractionation of plasma from male 16-week-old Aldoc^−/−^ and Aldoc^+/+^ littermate control mice fasted for four hours. (F) Plasma and (G) liver concentrations of cholesterol and triglycerides in female 16-week-old Aldoc^−/−^ and Aldoc^+/+^ littermate control mice fasted for four hours. H: Analysis of cholesterol-containing lipoproteins by FPLC fractionation of plasma from female 16-week-old Aldoc^−/−^ and Aldoc^+/+^ littermate control mice fasted for four hours. Data presented as mean ± SD. Statistical differences were determined with an unpaired two-tailed *t* test denoted by ∗*P* < 0.05, ∗∗*P* < 0.01, and ∗∗∗*P* < 0.001. Aldoc, aldolase C; FPLC, fast-protein liquid chromatography; Hmgcr, 3-hydroxy-3-methylglutaryl-CoA reductase; Hmgcs1, hydroxymethylglutaryl-CoA synthase 1.

To follow-up our in vitro study of *Aldoc* and its role in cholesterol metabolism, we investigated the physiological role of *Aldoc* in regulating cholesterol metabolism. We first tested the in vivo transcriptional regulation of liver *Aldoc*. Consistent with regulation by SREBP2, cholesterol feeding in mice significantly reduces the expression of liver *Aldoc* and the SREBP2 target gene, *Hmgcr*, and does not alter the expression of *Aldoa* or *Aldob* (Fig. 5A). Furthermore, hepatic expression of *Aldoc* and the SREBP2 target gene, *Hmgcs1* are significantly upregulated in fed mice compared to fasted mice, while *Aldoa* and *Aldob* expression are significantly reduced in the fed state (Fig. 5B). To test the physiological role for *Aldoc* in regulating cholesterol metabolism, we obtained a whole-body KO mouse for *Aldoc*. In male *Aldoc* KO (*Aldoc*^−/−^) mice, there was a significant reduction in plasma TC with no observed differences in plasma triglycerides or liver TC and triglyceride concentrations (Fig. 5C, D). FPLC fractionation of plasma identified the reduction of TC was due to a decrease in HDL-C in male *Aldoc*^−/−^ mice (Fig. 5E). In female *Aldoc*^−/−^ mice, we observed a significant reduction in plasma TC and triglyceride levels with no observed changes in liver TC and triglyceride concentrations (Fig. 5F, G). FPLC fractionation of plasma identified the reduction of TC was due to a decrease in HDL-C levels in female *Aldoc*^−/−^ mice (Fig. 5H). These data demonstrate that *Aldoc* has a role in regulating whole-body lipid metabolism in both male and female mice and is strongly regulated within the liver in a SREBP2-dependent manner.

**Fig. 6:**
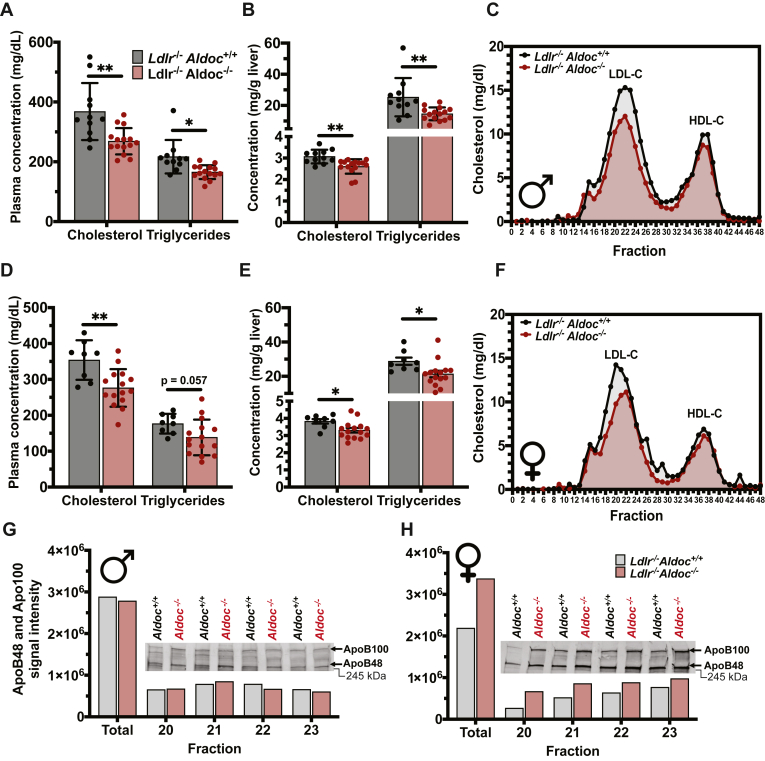
Loss of Aldoc reduces LDL-C in hyperlipidemic LDL receptor KO mice. (A) Plasma and (B) liver concentrations of cholesterol and triglycerides in male 12-week-old Ldlr^−/−^Aldoc^−/−^ and Ldlr^−/−^Aldoc^+/+^ littermate control mice fasted for four hours. C: Analysis of cholesterol-containing lipoproteins by FPLC fractionation of plasma from male 12-week-old Ldlr^−/−^Aldoc^−/−^ and Ldlr^−/−^Aldoc^+/+^ littermate control mice fasted for four hours. (D) Plasma and (E) liver concentrations of cholesterol and triglycerides in female 12-week-old Ldlr^−/−^Aldoc^−/−^ and Ldlr^−/−^Aldoc^+/+^ littermate control mice fasted for four hours. F: Analysis of cholesterol-containing lipoproteins by FPLC fractionation of plasma from female 12-week-old Ldlr^−/−^Aldoc^−/−^ and Ldlr^−/−^Aldoc^+/+^ littermate control mice fasted for four hours. (G) Male and (H) female ApoB48 and ApoB100 abundance in the indicated LDL-C associated FPLC fractions from Ldlr^−/−^Aldoc^−/−^ and Ldlr^−/−^Aldoc^+/+^ littermate control mice. Data presented as mean ± SD. Statistical differences were determined with an unpaired two-tailed *t* test denoted by ∗*P* < 0.05 and ∗∗*P* < 0.01. ApoB, apolipoprotein B; FPLC, fast-protein liquid chromatography; Ldlr, low-density lipoprotein receptor.

Mice and humans have very different distributions of plasma cholesterol between LDL-C and HDL-C with mice carrying a majority of their plasma cholesterol on HDL particles while humans are the opposite. *Ldlr* KO (*Ldlr*^−/−^) mice have a more human-like distribution of plasma cholesterol as they have a much slower clearance of LDL-C (39). We crossed *Aldoc*^−/−^ mice with *Ldlr*^−/−^ mice to determine if *Aldoc*^−/−^ mice would have reduced LDL-C on the *Ldlr*^*−/−*^ background. In male *Ldlr*^−/−^*Aldoc*^−/−^ mice fed a chow diet we observed a significant reduction in both plasma and liver TC and triglyceride concentration (Fig. 6A, B). FPLC fractionation of plasma indicated that this reduction in TC was a result of reduced LDL-C levels (Fig. 6C) and a decrease in triglyceride-rich lipoproteins in male *Ldlr*^−/−^*Aldoc*^−/−^ (Supplemental Fig. S4A). In female *Ldlr*^−/−^*Aldoc*^−/−^ mice fed a chow diet we also observed a significant reduction in plasma and liver TC and triglyceride levels (Fig. 6D, E). FPLC fractionation indicated this reduction in plasma TC and triglyceride levels was due to a decrease in plasma LDL-C (Fig. 6F) and triglyceride-rich lipoproteins in female *Ldlr*^*−/−*^*Aldoc*^*−/−*^ mice (Supplemental Fig. S4B). Analysis of ApoB abundance at the apex of the LDL-C peak and within the triglyceride-rich lipoproteins indicated that both male and female *Ldlr*^−/−^*Aldoc*^−/−^ mice have similar amounts of circulating LDL particles as their control, *Ldlr*^−/−^*Aldoc*^+/+^ littermates (Fig. 6G, H; Supplemental Fig. S4C–E). Body weight and liver weight in male *Ldlr*^*−/−*^*Aldoc*^*−/−*^ mice were significantly decreased, but the liver weight to body weight ratio was not significantly altered (Supplemental Fig. S4F–H). Collectively, these results establish an in vivo role for *Aldoc* in regulating liver and plasma cholesterol and triglyceride levels that are independent of uptake through the LDL receptor.

### Regulation of plasma lipid metabolism by hepatic *Aldoc*

**Fig. 7:**
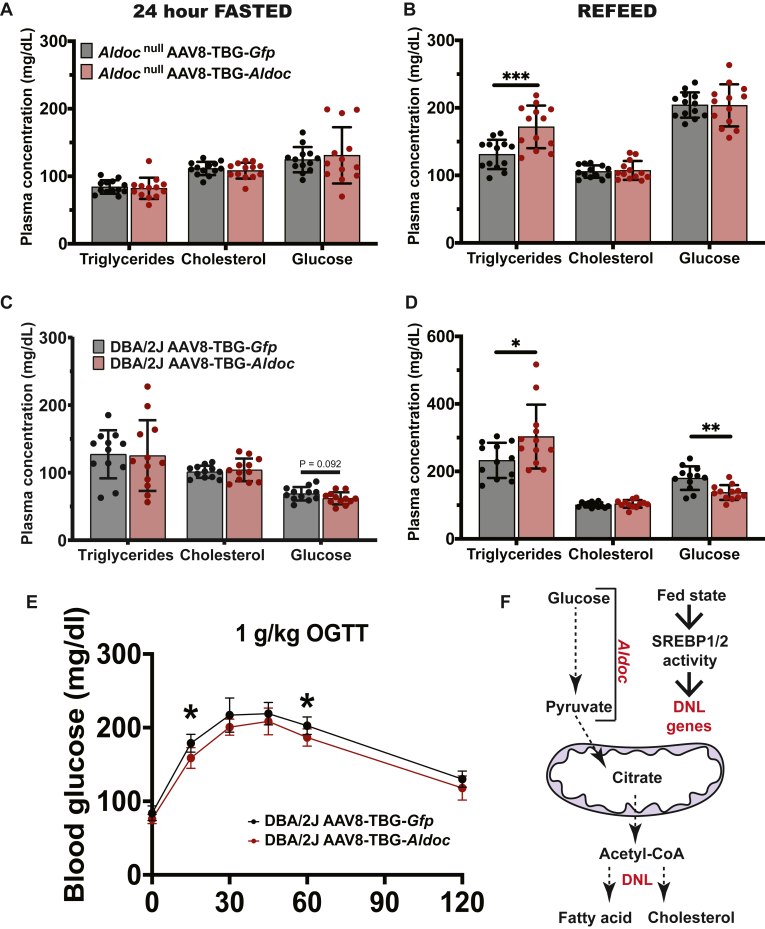
Liver-specific expression of Aldoc contributes to de novo lipogenesis. Plasma triglycerides, cholesterol, and glucose concentrations in male Aldoc^−/−^ mice treated with AAV8-TBG-*Gfp* or AAV8-TBG-*Aldoc* after either (A) a 24-h fast or (B) an overnight 14-h refeed with high-carbohydrate (70% sucrose) diet. Plasma triglycerides, cholesterol, and glucose concentrations in male DBA/2J mice treated with AAV8-TBG-*Gfp* or AAV8-TBG-*Aldoc* after either (C) a 24-h fast or (D) an overnight 14-h refeed with high-carbohydrate (70% sucrose) diet. E: Oral glucose tolerance test (1 g/kg glucose) in DBA/2J mice treated with either AAV8-TBG-*Gfp* or AAV8-TBG-*Aldoc*. F: Schematic model of overexpression of *Aldoc* leading to increased plasma triglycerides after refeeding with a 70% sucrose diet and activating liver de novo lipogenesis. Data presented as mean ± SD. Statistical differences were determined with an unpaired two-tailed *t* test denoted by ∗*P* < 0.05, ∗∗*P* < 0.01, and ∗∗∗*P* < 0.001. AAV8, adeno-associated virus 8; Aldoc, aldolase C; GWAS, genome-wide association studies; TBG, thyroxine-binding globulin.

To determine if *Aldoc* has a liver-specific role in modulating plasma cholesterol and triglycerides, we utilized a gain-of-function approach with AAV8 under the control of the TBG promoter to restore expression of *Aldoc* to hepatocytes of Aldoc^−/−^ mice. Mice were treated with AAV8-TBG-*Gfp* or AAV8-TBG-*Aldoc* and were fasted for 24 h and refed with a high carbohydrate diet for 16 h to strongly induce de novo lipogenesis and SREBP1 and SREBP2 signaling within the liver (40). In the fasted Aldoc^−/−^ mice, hepatic expression of *Aldoc* had no effect on plasma levels of triglycerides, cholesterol, or glucose (Fig. 7A). However, in high-carbohydrate refed *Aldoc*^*−/−*^ mice, hepatic expression of *Aldoc* resulted in a significant increase in plasma triglycerides (Fig. 7B). These data support a liver-specific role of *Aldoc* in the regulation of plasma triglyceride levels and de novo lipogenesis during the fed state.

We further tested the role of hepatic *Aldoc* in a mouse strain with increased hepatic de novo lipogenesis, the DBA/2J inbred mouse strain (41). In fasted male DBA/2J mice, hepatic expression of *Aldoc* with AAV8-TBG-*Aldoc* had no effect on plasma levels of triglycerides, cholesterol, or glucose (Fig. 7C). However, in high-carbohydrate refed male DBA/2J mice, hepatic expression of *Aldoc* resulted in a significant increase in plasma levels of triglycerides and a significant decrease in plasma glucose levels with no difference in plasma cholesterol levels (Fig. 7D). The reciprocal decrease in plasma glucose and increase in plasma triglycerides suggests that hepatic *Aldoc* may promote de novo lipogenesis through accelerated glucose utilization. To test if hepatic *Aldoc* can affect hepatic glucose uptake, we performed an oral glucose tolerance test in DBA/2J mice and observed significantly decreased glucose levels 15 and 60 min after glucose administration (Fig. 7E). Taken together, these experiments provide physiological evidence that hepatic *Aldoc* links carbohydrate metabolism to fatty acid metabolism in the fed state (Fig. 7F).

### Overlap with human liver coexpression networks

From our module-based coexpression network analysis of 35 genome-wide liver datasets, we were able to identify a reproducible module of genes significantly enriched for cholesterol biosynthetic genes across all datasets. The strong conservation of this module is likely due to the tight transcriptional regulation of genes involved in cholesterol metabolism within the liver through the action of SREBP1 and SREBP2 transcription factors. To determine if our approach would translate to human liver samples, we integrated our data with the Stockholm–Tartu Atherosclerosis Reverse Network Engineering Task (STARNET) study that measured genome-wide transcript levels using RNA sequencing in 600 coronary artery disease patients and 250 coronary artery disease-free control human liver samples (42). In the STARNET study, they performed module-based coexpression network analysis using WGCNA and identified a module significantly enriched for cholesterol biosynthesis genes. This human liver cholesterol module contains 59 protein-coding genes, of which many participate in cholesterol biosynthesis. From this human cholesterol module in STARNET that contained 59 genes, 49 of them overlapped with genes found in the mouse cholesterol modules (83% overlap) (Supplemental Table S4). From our replication analysis, 25 genes replicated more than ten times, and 24 genes were replicated from one to ten times. Importantly, two of the genes (*Rdh11* and *Aldoc*) that we prioritized for functional validation studies are contained within this human cholesterol module providing cross-species evidence that these two genes are relevant in liver cholesterol metabolism within humans.

### *Aldoc* is associated with human plasma cholesterol levels

**Fig. 8:**
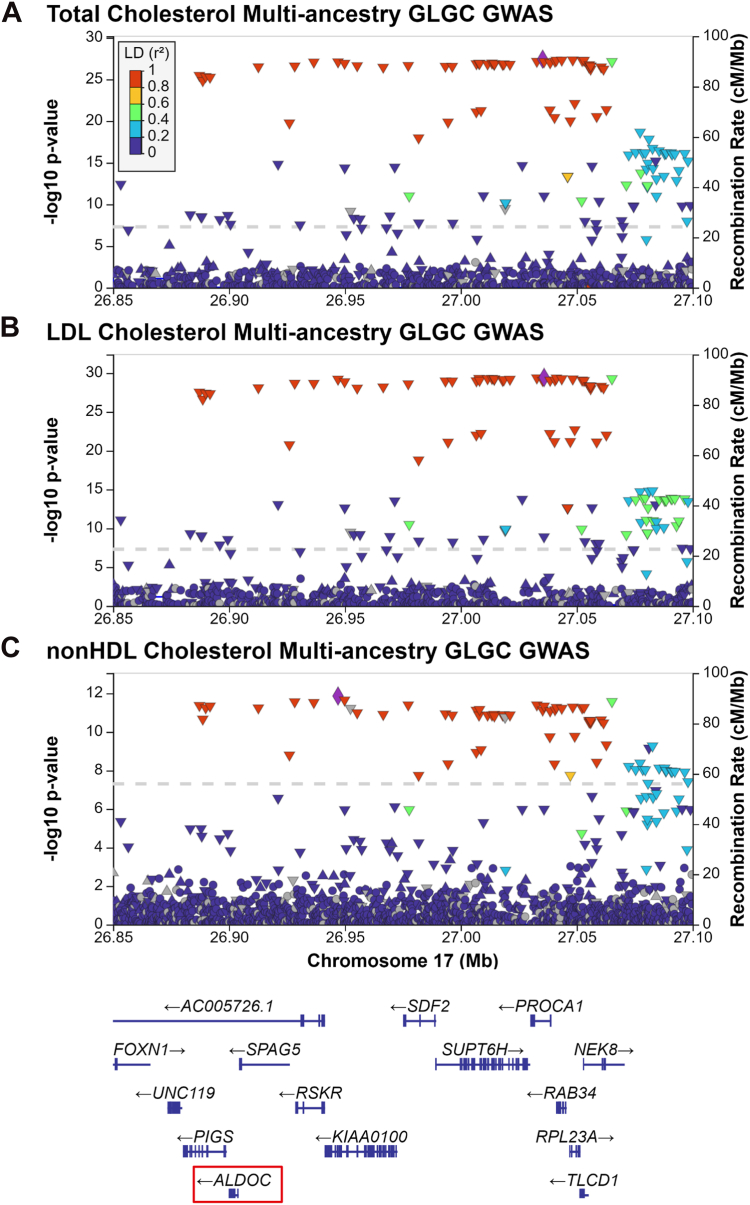
ALDOC is associated with lipid traits in human GWAS. Regional association plots of ALDOC with (A) total cholesterol, (B) LDL-cholesterol, and (C) nonHDL-cholesterol in the Global Lipid Genetics Consortium (GLGC) 2021 dataset of 1.65 million individuals. The left *y*-axis shows the significance of the association, and the right *y*-axis shows the recombination rate across the region (*blue line*). The *purple diamond* indicates the most associated single nucleotide polymorphism (SNP) in the region, and adjacent SNPs are colored according to the level of linkage disequilibrium (LD) with the most associated SNP. GWAS, genome-wide association studies; LDL-C, low density lipoprotein cholesterol.

In previous work, we demonstrated that liver module-based coexpression networks in the mouse can be used to prioritize genes within human lipid GWAS loci (12). To expand this approach, we performed a systematic analysis of 35 mouse liver expression datasets and were able to identify and validate the role of three genes (*Rdh11*, *Echdc1*, and *Aldoc*) in cholesterol metabolism. To determine if these three genes, which had no prior known role in cholesterol metabolism contributed to variation in human plasma cholesterol levels, we cross-referenced these genes with a recent multiancestry human GWAS of 1.65 million people for TC, LDL-Cl, nonHDL-C, and triglyceride levels (5). From this analysis, we identified that *Aldoc* is located within a genome-wide significant locus on chromosome 17 associated with TC (rs16963468; *P* = 1.68 × 10^−27^), LDL-cholesterol (rs9915479; *P* = 3.81 × 10^−30^), and nonHDL-cholesterol (rs2302205; *P* = 1.71 × 10^−14^) levels in humans (Fig. 8A–C) (5). This locus contains 12 protein-coding genes within the region of strong linkage disequilibrium, making it a challenging locus to identify a causal gene. Based on our functional validation studies, *Aldoc* is likely a causal gene within this locus.

## Discussion

Technological advances in our ability to quantitate genome-wide gene expression and protein levels have paved the way for tremendous insights into biological systems. Systems genetics studies have capitalized on these advances and used genome-wide gene expression and protein data from diverse genetic backgrounds in mice and humans to understand the genetic architecture of complex traits (15, 18, 43). In our previous work, we demonstrated the feasibility of module-based coexpression network analysis across 12 genome-wide mouse liver datasets to identify candidate causal genes located within human lipid GWAS loci (12). Here, we extend this approach with the goal of exclusively using module-based network analysis to identify cholesterol metabolism-related genes from a comprehensive catalog of 35 datasets. From our analysis, we were able to identify 36 genes present within the cholesterol module replicated in more than 10 datasets. Within these 36 core genes, 32 of the genes are bona fide genes involved in regulating cholesterol metabolism. The most replicated of the remaining genes, *Rdh11*, *Echdc1*, *Aldoc*, and *Paox* had no known role in cholesterol metabolism and the top three (*Rdh11*, *Echdc1*, and *Aldoc*) were prioritized for functional validation studies based on regulation by SREBP2.

The first gene we prioritized for validation studies was *Rdh11* (replicated 24 times out of 35 datasets). *Rdh11* is a member of the short-chain dehydrogenase/reductase family of proteins that converts retinaldehyde to retinol and is capable of the reduction of short-chain aldehydes (35, 44). Our validation studies demonstrated hepatic *Rdh11* is transcriptionally regulated by cholesterol levels through SREBP2. Through functional studies, we demonstrated that *Rdh11* knockdown results in increased cellular cholesterol when cholesterol biosynthesis is upregulated by lipoprotein-deficient serum treatment. These results establish that *Rdh11* plays a role in regulating cellular cholesterol metabolism within cells and validates our systematic prioritization of *Rdh11*.

The second gene we prioritized for validation was *Echdc1* (replicated 13 times out of 35 datasets). *Echdc1* is a cytosolic enzyme that mediates the decarboxylation of ethyl-malonyl-CoA and methyl-malonyl-CoA (45). Previous work has proposed that *Echdc1* acts as a proof-reading enzyme to limit the incorporation of ethyl-malonyl-CoA and methyl-malonyl-CoA into fatty acids, which would generate branched-chain fatty acids during fatty acid synthesis (45). Consistent with this role genetic ablation of *Echdc1* both in vitro and in vivo increases the prevalence of branched-chain fatty acids (46, 47). Our validation studies show that *Echdc1* is transcriptionally regulated by cholesterol through the action of SREBP2. Using a whole-body KO mouse for *Echdc1*, we demonstrated that *Echdc1* KO mice on a standard chow diet have elevated plasma levels of cholesterol and triglycerides. Fractionation of plasma by FPLC revealed a specific increase in LDL-C and triglycerides within the VLDL fractions in *Echdc1* KO mice. Taken together, our functional validation studies establish *Echdc1* as a gene capable of regulating plasma cholesterol and triglyceride metabolism.

The third gene we prioritized for validation was *Aldoc*, one of the three aldolase isozymes, which differ based on protein and DNA sequence homology, catalytic activities, relative tissue expression patterns, and genomic location (38). Enzymatically, the aldolases act in the glycolysis and gluconeogenesis pathways to carry out the reversible cleavage of F1,6-bisP to DHAP and glyceraldehyde 3-phosphate. We established that hepatic *Aldoc* is the only aldolase isozyme regulated in a SREBP2 dependent manner. We also established that *Aldoc* has a role in the regulation of cholesterol metabolism. In AML12 mouse hepatocytes, *Aldoc* is necessary for the efficient conversion of glucose, but not acetate, into cholesterol and the maintenance of cellular cholesterol concentrations when cholesterol is depleted from cell. Further analysis showed *Aldoc* knockdown impacts glucose metabolism and cholesterol biosynthesis without influencing total aldolase activity and its canonical substrates F1,6-bisP or DHAP, suggesting an enzyme-independent role for *Aldoc*. Research has shown that the related aldolase, *Aldoa*, when unbound from F1,6BP (low glucose conditions) can signal and activate lysosomal AMP kinase (48, 49, 50), and *Aldoa* can act as a protein scaffold and bind to the actin cytoskeleton (51, 52). It is possible that *Aldoc* may have a similar regulatory role beyond its glycolytic enzyme function, which is an important direction for future research. To establish an in vivo role for *Aldoc* in the regulation of cholesterol metabolism, we utilized three distinct mouse models including whole-body *Aldoc* KO mice, hypercholesterolemic *Ldlr* KO mice, and hepatic specific expression of *Aldoc*. Through the use of these three models, we were able to demonstrate that Aldoc has a significant impact on plasma and hepatic cholesterol and triglyceride metabolism.

In summary, our studies demonstrate the utility and power of module-based coexpression networks in identifying a module of genes involved in cholesterol metabolism. We also show that this module of genes identified among mouse liver expression datasets is conserved and present in human liver samples. Through our systematic analysis of 35 genome-wide mouse liver expression datasets, we were able to identify, prioritize, and validate three genes (*Rdh11*, *Echdc1*, and *Aldoc*) that had no prior known role in cholesterol metabolism. For *Aldoc*, we provide detailed biochemical characterization and show *Aldoc* is located within a genome-wide significant locus associated with plasma levels of TC, LDL-C, and nonHDL-C in human GWAS. Our data indicate that *Aldoc* is a causal gene within this locus and contributes to variation in plasma cholesterol levels through regulating de novo cholesterol biosynthesis. These studies establish a role for *Aldoc* in linking carbohydrate metabolism to lipid metabolism. Collectively, we were able to take advantage of the wealth of publicly available mouse genome-wide liver expression datasets and the intricately regulated transcriptional network that controls cholesterol metabolism to identify new modifiers of this biochemical process.

## Data availability

Genome-wide transcript and protein data used for WGCNA analysis can obtained from indicated Gene Expression Omnibus (GEO, NCBI) public repository with indicated accession numbers found in Supplemental Table S1. All remaining data are contained within the article.

## Supplemental data

This article contains supplemental data (16, 17, 18, 19, 21, 22, 24, 25, 27, 28, 30).

## Conflict of interest

The authors declare that they have no conflicts of interest with the contents of this article.
