# Supplementary material for: Mining cholesterol genes from thousands of mouse livers identifies aldolase C as a regulator of cholesterol biosynthesis
Source: J Lipid Res. 2024 Feb 28;65(3):100525. doi: 10.1016/j.jlr.2024.100525 (PMC10965479; doi:10.1016/j.jlr.2024.100525)
Supplement: Supplemental Figure S1 [file mmc1.pdf]

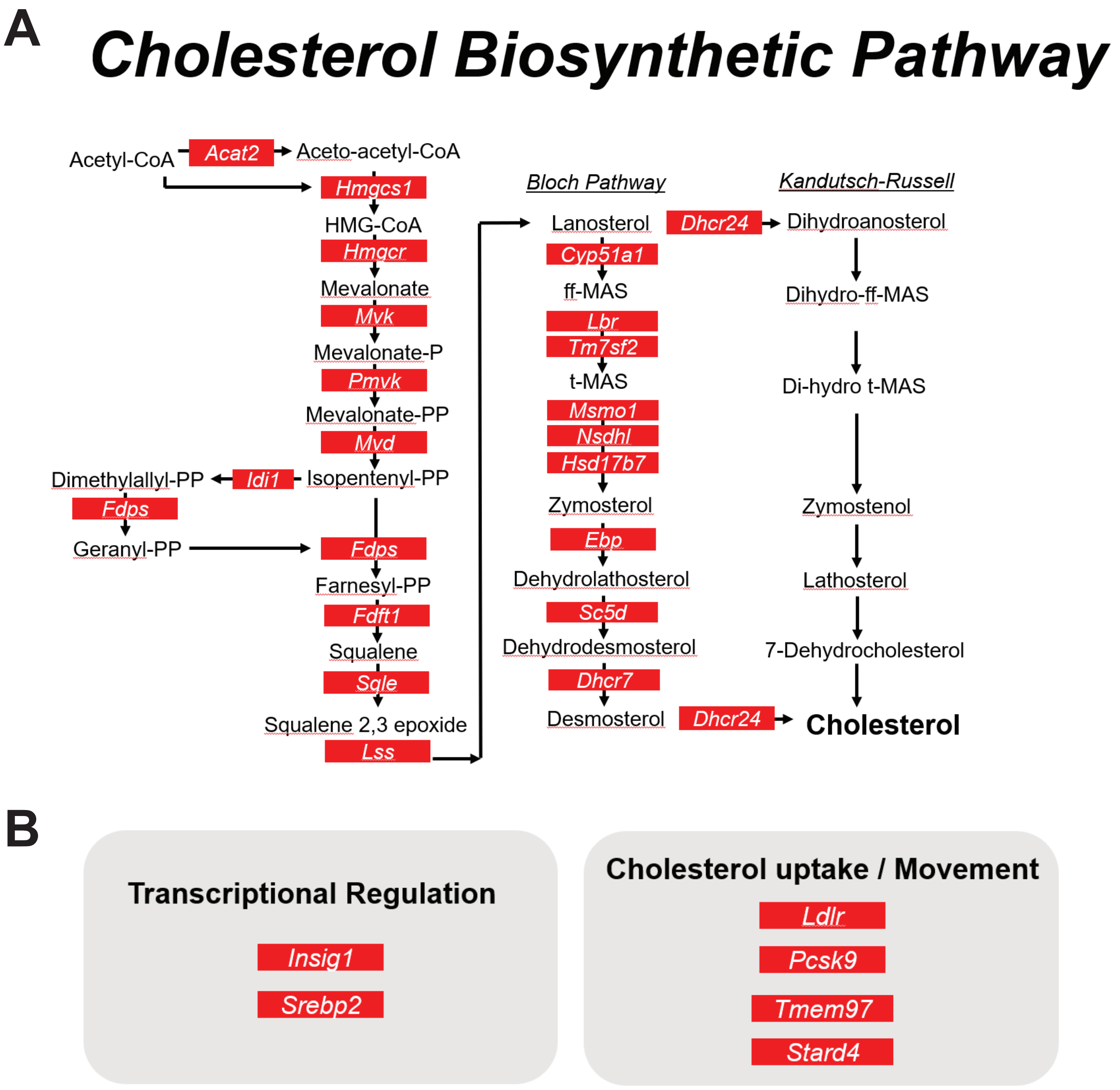

Supplemental Figure 1: Identification of complete complement of genes involved in cholesterol metabolism.

(A) Schematic of the cholesterol biosynthetic pathway from Acetyl-CoA ending in cholesterol with highlighted genes in red that where replicated more than 10 times within the cholesterol modules across the 35 mouse liver genome-wide expression datasets. **(B)** Genes involved in transcriptional regulation of cholesterol and cholesterol uptake/movement that were identified among the genes replicated more than 10 times within the cholesterol modules across the 35 mouse liver genome-wide expression datasets.
