# Supplementary material for: Mining cholesterol genes from thousands of mouse livers identifies aldolase C as a regulator of cholesterol biosynthesis
Source: J Lipid Res. 2024 Feb 28;65(3):100525. doi: 10.1016/j.jlr.2024.100525 (PMC10965479; doi:10.1016/j.jlr.2024.100525)
Supplement: Supplemental Figure S2 [file mmc2.pdf]

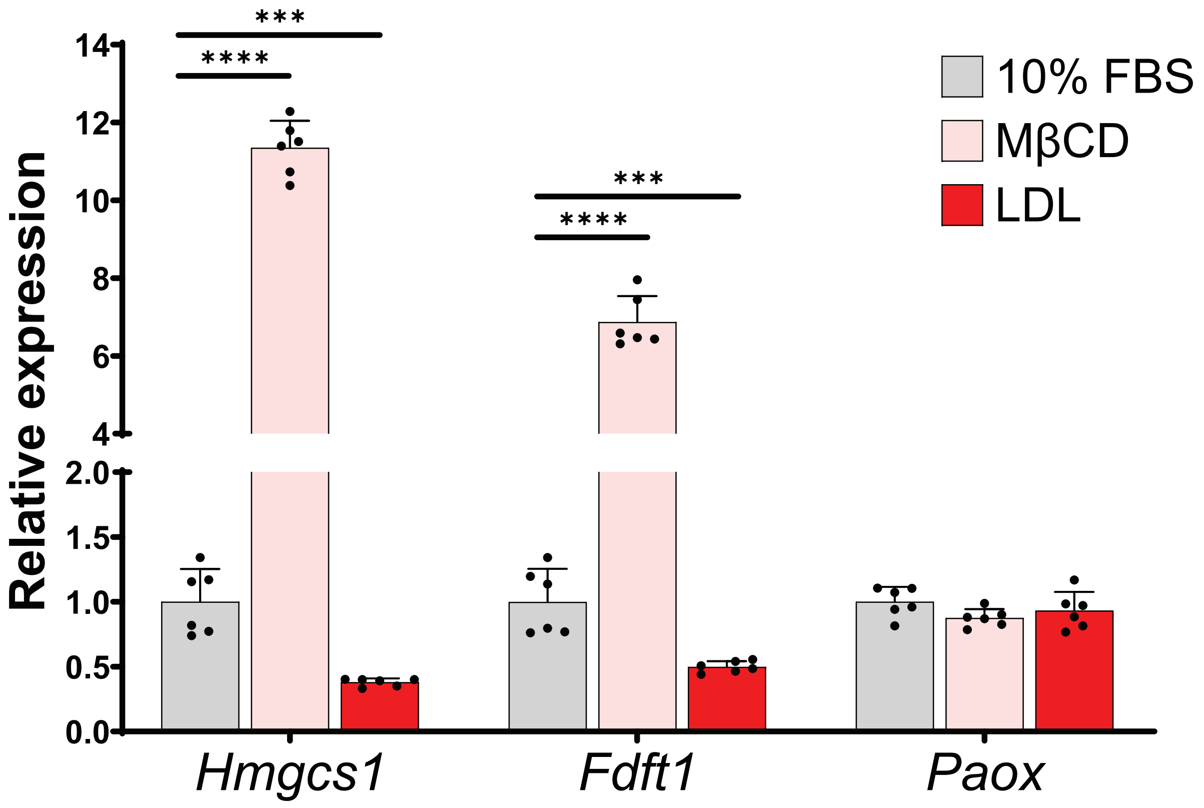

Supplemental Figure 2: Paox is not regulated by cholesterol depletion or supplementation in cells.

Relative mRNA expression of *Hmgcs1, Fdft1,* and *Paox*, in mouse AML12 hepatocyte cells incubeted with 10% FBS, MβCD, or 100 µg/mL human LDL-C for 16 hours. Data presented as mean ± SD. Statistical differences were determined with an unpaired two-tailed t-test denoted by ***P < 0.001, and ****P < 0.0001.
