# Supplementary material for: Mining cholesterol genes from thousands of mouse livers identifies aldolase C as a regulator of cholesterol biosynthesis
Source: J Lipid Res. 2024 Feb 28;65(3):100525. doi: 10.1016/j.jlr.2024.100525 (PMC10965479; doi:10.1016/j.jlr.2024.100525)
Supplement: Supplemental Figure S3 [file mmc3.pdf]

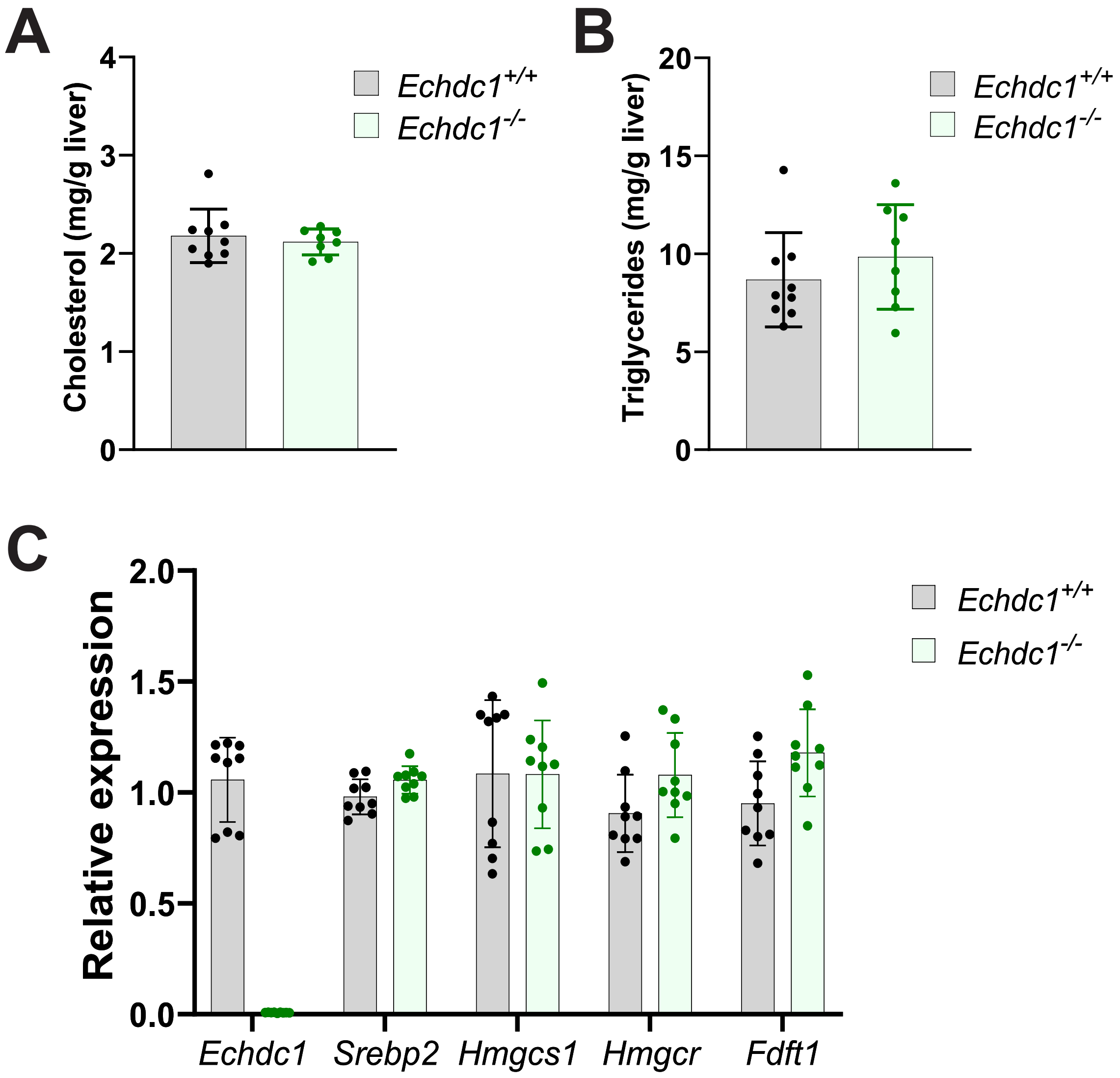

Supplemental Figure 3: Echdc1 deletion in mice does not influence hepatic lipid stores or expression of cholesterol genes.

Liver concentration of **(A)** cholesterol and **(B)** triglycerides in male 8-week-old Echdc1$^{-/-}$ and Echdc1$^{+/+}$ littermate control mice fasted for four hours. **(C)** Relative liver mRNA expression of *Echdc1*, *Srebp2*, *Hmgcs1*, *Hmgcr*, and *Fdft1* in male 8-week-old Echdc1$^{-/-}$ and Echdc1$^{+/+}$ littermate control mice fasted for four hours. Data presented as mean ± SD.
