# Supplementary material for: Mining cholesterol genes from thousands of mouse livers identifies aldolase C as a regulator of cholesterol biosynthesis
Source: J Lipid Res. 2024 Feb 28;65(3):100525. doi: 10.1016/j.jlr.2024.100525 (PMC10965479; doi:10.1016/j.jlr.2024.100525)
Supplement: Supplemental Figure S4 [file mmc4.pdf]

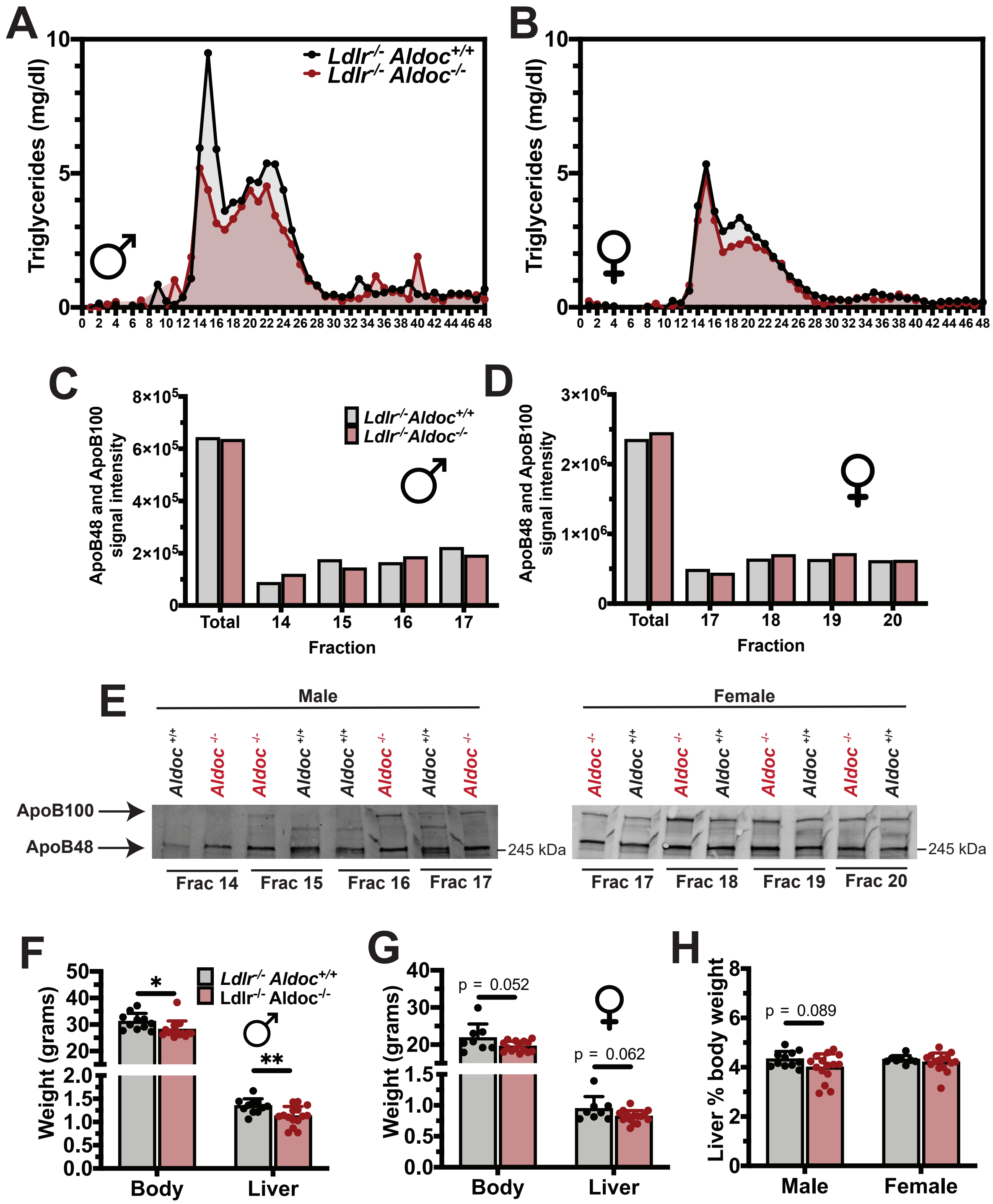

Supplemental Figure 4. Loss of *Aldoc* in LDL receptor knockout mice reduces plasma lipid levels.

Triglyceride concentration in FPLC fractions of **(A)** male and **(B)** female $Ldlr^{-/-}Aldoc^{-/-}$ and $Ldlr^{-/-}Aldoc^{+/+}$ mice. ApoB48 and ApoB100 abundance in the indicated triglyceride-associated FPLC fractions of **(C)** male and **(D)** female $Ldlr^{-/-}Aldoc^{-/-}$ and $Ldlr^{-/-}Aldoc^{+/+}$ mice. **(E)** ApoB western blots of indicated triglyceride-associated FPLC fractions in male and female $Ldlr^{-/-}Aldoc^{-/-}$ and $Ldlr^{-/-}Aldoc^{+/+}$ mice. Bodyweight and liver weight of 12-week-old **(F)** male and **(G)** female $Ldlr^{-/-}Aldoc^{-/-}$ and $Ldlr^{-/-}Aldoc^{+/+}$ mice. **(H)** Liver weight as a percent of body weight for male and female mice. Data presented as mean ± SD. Statistical differences were determined with an unpaired two-tailed t-test denoted by *P < 0.05 and **P < 0.01.
